# Synthesis and Antioxidant Activity of Some New Coumarinyl-1,3-Thiazolidine-4-ones 

**DOI:** 10.3390/molecules15106795

**Published:** 2010-09-29

**Authors:** Milan Čačić, Maja Molnar, Bojan Šarkanj, Elizabeta Has-Schön, Valentina Rajković

**Affiliations:** 1 Department of Applied Chemistry and Ecology, Faculty of Food Technology, J. J. Strossmayer University, Franje Kuhača 20, 31 000 Osijek, Croatia; 2 Department of Biology, J. J. Strossmayer University, Trg Ljudevita Gaja 6, 31 000 osijek, Croatia

**Keywords:** coumarin, hydrazides, aromatic Schiff^’^s bases, *N*-(2-aryl-4-oxo-thiazolidin-3-yl)-2-(4-methyl-2-oxo-2*H*-chromen-7-yloxy)acetamide, antioxidant activity, biological activity

## Abstract

A series of Schiff’s bases (*E*)-*N*-2-aryliden-2-(4-methyl-2-oxo-2*H*-chromen-7-yloxy)acetohydrazides **2a-l** and *N*-(2-(substituted phenyl)-4-oxo-thiazolidin-3-yl)-2-(4-methyl-2-oxo-2*H*-chromen-7-yloxy)acetamides **3a-l** were synthesized and evaluated for their antioxidant activity by the phosphomolybdenum method. Most of the Schiff’s bases and thiazolidine-4-ones bearing two hydroxyl groups on the phenyl ring showed excellent antioxidant activity in comparison with ascorbic acid. Preliminary investigation on cytotoxic and antifungal activity was done on some representative samples.

## 1. Introduction

The structural and therapeutic diversity of small heterocyclic molecules coupled with their commercial viability has long fascinated organic and medicinal chemists. Heterocycles containing the coumarin ring system include some novel pharmacologically active compounds such as dicumarol, warfarin, mercumatilin and novobiocin. Natural coumarins affect the formation and scavenging of ROS and influence free radical-mediated oxidative damage [1].

Azomethine group (-C=N-)-containing compounds, typically known as Schiff’s bases, have been synthesized via condensation of primary amines with active carbonyls. It is well established that the biological activity of hydrazone compounds is associated with the presence of the active (-CO-NH-N=C-) pharmacophore and these compounds form a significant category of compounds in medicinal and pharmaceutical chemistry with several biological applications that include antitumoral [2,3], antifungal [4,5,6,7,8,9], antibacterial [4,5,6,7,8,9,10,11], antimicrobial [12] and anthelmintic uses [13]. Schiff’s base complexes play an important role in designing metal complexes related to synthetic and natural oxygen carriers [14]. 

In recent years, 4-thiazolidinones and 2,4-thiazolidinediones have been among the most extensively investigated classes of organic compounds. Thiazolidine derivatives are reported to show a variety of biological activities. The presence of a thiazolidine ring in penicillin and related derivatives was the first recognition of its occurrence in nature [15]. Thiazolidine-4-one represents a prevalent scaffold in drug discovery [16]. Literature surveys show that thiazolinylhydrazones exhibit antitubercular and antimicrobial activities [15], and their pronounced antioxidant [17] and antifungal [18] activity has also been reported. Thiazolidine-4-ones have many interesting activity profiles, namely COX-1 inhibitors [19], inhibitors of the bacterial enzyme MurB, which was a precursor acting during the biosynthesis of peptidoglycan [20], non-nucleoside inhibitors of HIV-RT [21] and anti-histaminic agents [22]. Depending on the substituents, 4-oxothiazolidine ring can induce different pharmacological properties such as antibacterial [23], antimycobacterial [24], anticonvulsant [25] or anti-inflammatory activity [26] and it has been reported that the introduction of arylidene moieties at different positions of the thiazolidinone ring enhanced biological activity [27,28,29]. Some authors examined the ability of this ligand structure to form complexes with some radionuclides for potential use in nuclear medicine [30]. Thus, coumarins containing a Schiff’s base and a thiazolidinone moiety are expected to have enhanced biological activities.

## 2. Results and Discussion

### 2.1. Synthesis

**Scheme 1:**
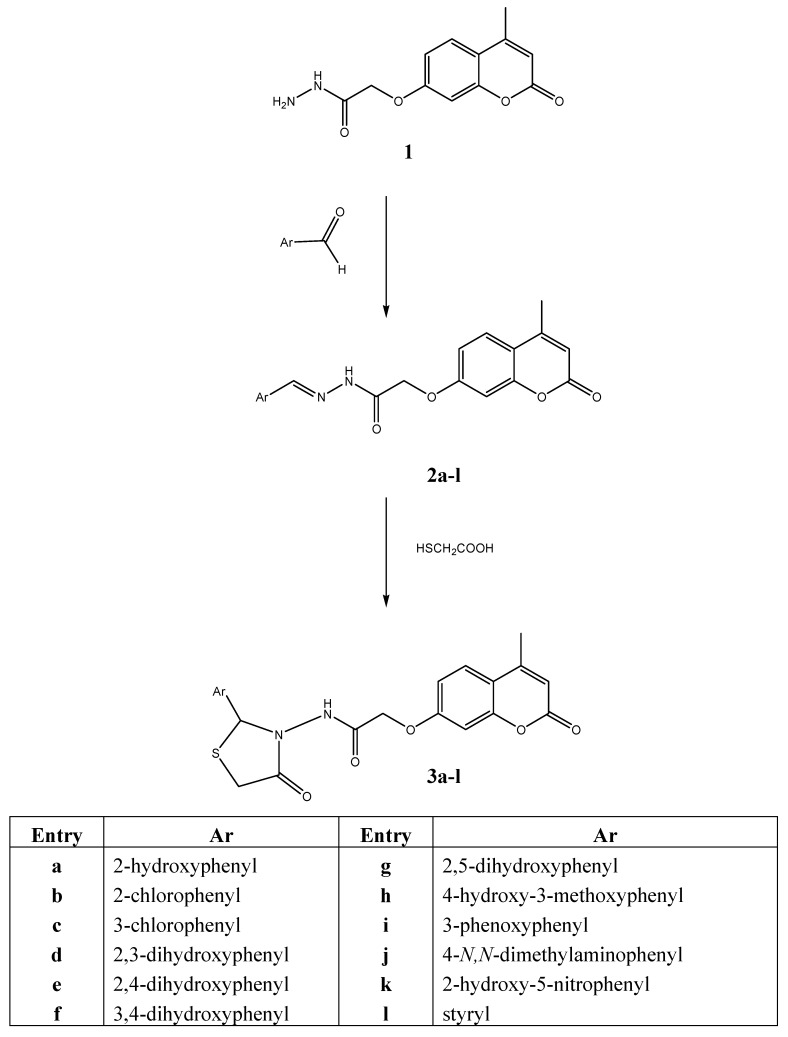
Synthesis of 2,3-disubstituted-1,3-thiazolidine-4-ones **3a-l**.

In our ongoing research to synthesize potentially biologically active thiazolidinone derivatives we have now described a series of (*E*)-*N*-2-aryliden-2-(4-methyl-2-oxo-2*H*-chromen-7-yloxy)aceto-hydrazides **2a-l** and *N*-(2-aryl-4-oxo-thiazolidine-3-yl)-2-(4-methyl-2-oxo-2*H*-chromen-7-yloxy)-acetamides **3a-l (Scheme 1)**. The series of Schiff^’^s bases **2a-l** was prepared similarly to those previously described [5,6,12,13,31], by refluxing solutions of different suitable aromatic aldehydes and hydrazide **1 **in absolute ethanol for 2 to 4 hours, in a presence of catalytic amount of glacial acetic acid. The structures of the products **2a-l **were inferred from their analytical and spectral data.

**Scheme 2:**
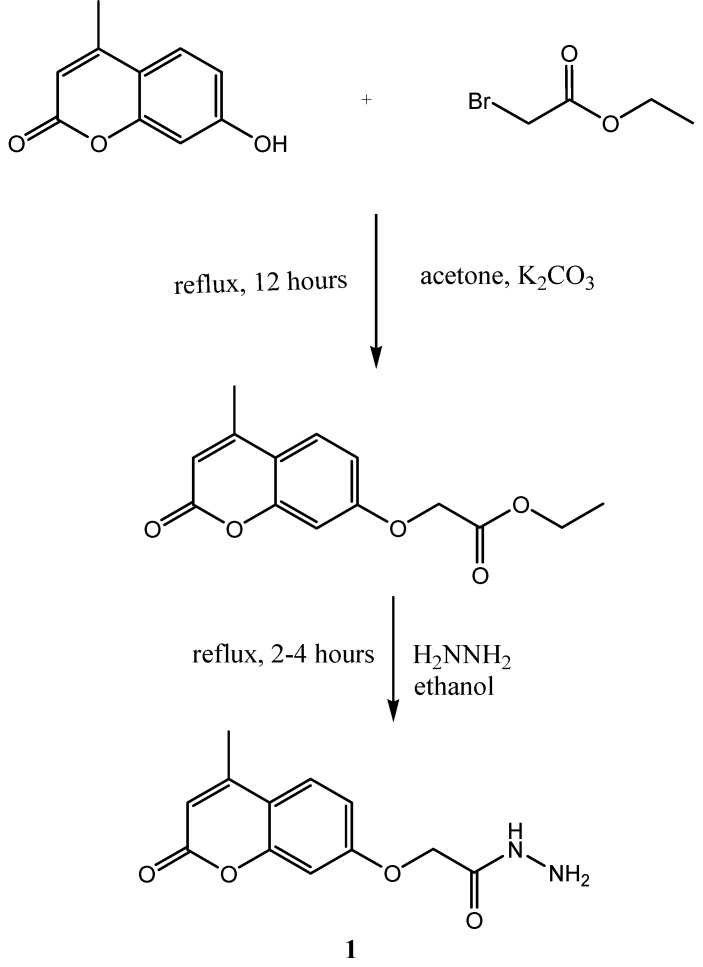
Synthesis of (4-methyl-2-oxo-2*H*-chromen-7-yloxy)acetic acid hydrazide **1**.

Starting material **1** was prepared as indicated in **Scheme 2.** Its IR spectrum showed absorption bands in the 3,317 cm^-1 ^(hydrazide NH-NH_2_), 3,269 cm^-1 ^(aromatic C-H), 1,711 cm^-1^(-C=O carbonyl stretching) and 1,621–1,640 cm^-1^ (-CO-NH-NH_2_ groups) regions, respectively. The ^1^H-NMR spectrum exhibited a singlet due to the –CO-NH-NH_2_, NH proton at δ 9.32 ppm. Methylene protons (-OCH_2_-) resonated as singlets at 4.85 ppm.

The IR spectra of compounds **2a-l** showed characteristic bands at 3,448–3,278 cm^-1^ (OH; NH), 1,709 cm^-1 ^and 1,672 cm^-1^(C=O, lactone) and 1,620 cm^-1^ (C=O, amide, HC=N azomethine). The ^1^H- NMR spectra did not only show the absence of NH_2_ protons at 3.38, but also the presence of N=CH proton at 8.30 ppm.

N-(2-aryl-4-oxo-thiazolidine-3-yl)-2-(4-methyl-2-oxo-2*H*-chromen-7-yloxy) acetamides **3a-l **were obtained by the reaction described in [29], which was performed by refluxing the solutions of Schiff’s bases **2a-l** and thioglycolic acid in 1,4-dioxane in the presence of anhydrous ZnCl_2_ for 6-8 hours. Formation of 2,3-disubstituted 4-thiazolidinones **3a-l** was confirmed by IR spectroscopy, which showed the ring C=O stretching characteristic of 1,3-thiazolidine-4-ones ring in the range of ν_max _1,690–1,730 cm^-1^.

^1^H-NMR spectra for **3a-l **showed methylene CH_2 _(COCH_2_S) protons of the 4-thiazolidinone ring between δ 3.34–3.38 ppm as the singlet signal and δ5.26–5.29 ppm for CH (SCHN); proton of the 4-thiazolidinone ring as a singlet signal.

### 2.2. Antioxidant activity

**Figure 1:**
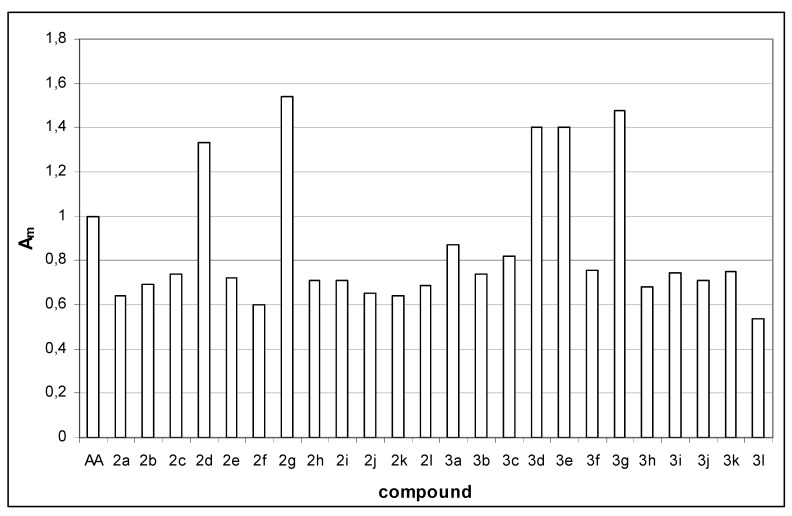
Antioxidant activities of novel coumarin derivatives relative to ascorbic acid (*A_m_* – activity relative to ascorbic acid (AA) on a molar basis).

Data in **Figure 1**. show that substituents on the phenyl ring have a great influence on antioxidant activity. In descending order the effects of the various substituents on the phenyl ring of the Schiff’s bases were found to be: 2,5(OH)_2_ (**2g**) > 2,3-(OH)_2 _(**2d**) > 3-Cl (**2c**) > 2,4-(OH)_2_ (**2e**) > 3 - phenoxy (**2i**) > 3-OCH_3_-4-OH (**2h**) > 2-Cl (**2b**) > styryl (**2l**) > 4-N(CH_3_)_2_ (**2j**) > 2-OH-5-NO_2_ (**2k**)> 2-OH (**2a**) > 3,4-(OH)_2_ (**2f**). Among the Schiff’s compounds **2g** and **2d** have better antioxidant activities than ascorbic acid (1.54 and 1.34 times better, respectively). Both of these compounds have two electron donating OH groups on phenyl ring, one of them being in *ortho* position in both cases. They also posses another electron donating group, the presence of which obviously contributes to increased antioxidant activity, as the compound **2a** with only one OH group in the *ortho* position did not show relevant antioxidant activity. 

The effects of various substituents on phenyl ring of 1,3-thiazolidine-4-ones in descending order were found to be: 2,5-(OH)_2_ (**3g**) > 2,3-(OH)_2_ (**3d**) > 2,4-(OH)_2_ (**3e**) > 2-OH (**3a**) > 3-Cl (**3c**) > 3,4-(OH)_2 _(**3f**) > 2-OH-5-NO_2_ (**3k**) > 3-phenoxy (**3i**) > 2-Cl (**3b**) > 4-N(CH_3_)_2_ (**3j**) > 3-OCH_3_-4-OH (**3h**) > styryl (**3l**). Among the series of 1,3-thiazolidine-4-ones, compounds **3g**, **3d** and **3e** have better antioxidant activity than ascorbic acid (1.48, 1.41 and 1.40 times better, respectively). All of these compounds also have two electron donating OH groups on the phenyl ring, one of them being in an *ortho* position. Presence of another OH group, no matter the position on the phenyl ring, obviously contributes to increased antioxidant activity, like in the series of Schiff’s bases.

Observing the overall data for antioxidant activity, it is clear that the presence of two hydroxyl groups has a great influence on radical scavenging activity. Schiff’s base **2g** shows the greatest antioxidant activity of all investigated compounds, followed by the 1,3-thiazolidine-4-one **3g, **both having 2,5-(OH)_2_ substituents on phenyl ring**, **which is in accordance with the results of Lin *et al*. [32] who reported correlation of radical-scavenging effects of coumarins with the number of hydroxyl groups. 

## 3. Experimental

### 3.1. General

The melting points were taken on an Electrothermal capillary melting point apparatus and are uncorrected. Thin-layer chromatographies were performed using HF_254 _fluorescent silica gel plates (Merck), which were examined under UV 254 and 365 nm light. Silica gel (230–400 mesh) was used for flash chromatography separations. The elemental analysis for C, H and N were done on a Perkin-Elmer Analyzer 2440. Infrared spectra (ν/cm^-1^) were recorded on a Beckmann FT-IR 3303 instrument, using KBr disks. ^1^H- and ^13^C-NMR spectra were recorded on JEOL EX-270 MHz NMR Spectrometer at 293 K in DMSO-d_6_. Spectra were internally referenced to TMS. Peaks are reported in ppm downfield of TMS. The absorbance was measured on a Helios γ UV visible spectrophotometer (Thermo Spectronic, Cambridge, UK).

### 3.2. General procedure for preparation of (E)-Ń’-arylidene-2-(4-methyl-2-oxo-2H-chromen-7-yloxy)-acetohydrazides *2a-l*

A mixture of compound **1 **(0.01 mole) and a suitable aromatic aldehyde (**Ar/a-k**; 0.01 mole) was refluxed in absolute ethanol (30 mL) in presence of a catalytic amount of glacial acetic acid for 2 to 4 hours. The reaction mixture was cooled and the precipitate was filtered and recrystallized from methanol to give compounds **2a-l**. 

*(E)-N^´’^-(2-Hydroxybenzylidene)-2-(4-methyl-2-oxo-2H-chromen-7-yloxy)acetohydrazide *(**2a**) [5]. M.p. 284–286 °C; yield (72%); FT-IR: ν_max_ 3,440; 3,282; 3,101; 2,916; 2,853; 1,724; 1,685; 1,621; 1,537; 1,489; 1,393; 1,300 and 1,154 cm^-1^. ^1^H-NMR (δ, ppm): 2.41(s, 3H, CH_3, _C-4); 4.85 (s, 2H, CH_2_); 6.25 (s, 1H, C-3); 6.87 (s, 1H, H-8), 6.98 (d, 1H, H-6); 7.85 (d, 1H, H-5); 7.25-7.75 (m, 4H, arom.,) 8.33 (s, 1H, NH); 8.56 (s, 1H, HC=N-); 10.99 (s,1H, OH). ^13^C-NMR (δ, ppm): 21.4 (CH_3_); 69.10 (OCH_2_); 107.65 (C-8); 111.0 (C-6); 112.8 (C-3); 113.5 (C-9); 116.1 (C-3, Ar-); 118.4 (C-3, Ar-); 121.7 (C-5, Ar-); 127.8 (C-5); 130.5 (C-6, Ar-); 132.4 (C-1, Ar-); 161.2 (C-2, Ar-); 143.2 (C=N); 151.4 (C-9); 152.9 (C-4); 160.3 (C-7); 160.9 (C-4); 172.42 (CO-NH). Anal. Calcd. for C_19_H_16_N_2_O_5_ (352.34): C, 64.77; H, 4.58; N, 8.95. Found: C, 64.76; H, 4.55; N, 7.92 

*(E)-N^´^-(2-Chlorobenzylidene)-2-(4-methyl-2-oxo-2H-chromen-7-yloxy)acetohydrazide* (**2b**) [13]. M.p. 248–250 °C; yield (82%). FT-IR: ν_max_ 3,289; 2,950; 2,864; 1,712; 1,700; 1,624; 1,560; 1,525; 1,398; 1,290 and 1,150 cm^-1^. ^1^H-NMR (δ, ppm): 2.40 (s, 3H, CH_3_, C-4); 4.84 (s, 2H, CH_2_); 6.22 (s, 1H, H-3); 6.99 (s, 1H, H-8); 7.05 (d, 1H, H-6); 7.39-7.75 (m, 4H, arom.); 8.06 (d, 1H, H-5); 8.41(s, 1H, NH); 8.74 (s, 1H, HC=N-). ^13^C-NMR (δ, ppm): 21.4 (CH_3_); 69.10 (OCH_2_); 107.65 (C-8); 111.0 (C-6); 112.8 (C-3); 113.5 (C-9); 127.1 (C-5, Ar-); 127.8 (C-5); 129.1 (C-3, Ar-); 130.4 (C-6, Ar‑); 132.5 (C-4, Ar-); 133.2 (C-1, Ar-); 134.2 (C-2, Ar-); 143.2 (C=N); 151.4 (C-9); 152.9 (C-4); 160.3 (C-7); 160.9 (C-2); 172.42 (CO-NH). Anal. Calcd. for C_19_H_15_ClN_2_O_4_ (370.79): C, 61.55; H, 4.08; N, 7.56. Found: C, 61.57; H, 4.08; N, 7.53. 

*(E)-N^´^-(3-Chlorobenzylidene)-2-(4-methyl-2-oxo-2H-chromen-7-yloxy)acetohydrazide* (**2c**) [5]. M.p. 242–244 °C; yield (80%). FT-IR: ν_max_ 3,448; 3,201; 3,102; 2,997; 1,713; 1,683; 1,617; 1,511; 1,434; 1,391; 1,274 and 1,136 cm^-1^. ^1^H-NMR (δ, ppm): 2.40 (s, 3H, CH_3, _C-4); 4.82 (s, 2H, CH_2_); 6.23 (s, 1H, H-3); 6.98 (s, 1H, H-8); 7.02 (d, 1H, H-6); 7.20 (d, 1H, H-5); 7.41-7.74 (m, 4H, arom.); 8.00 (s, 1H, NH); 8.31 (s, 1H, HC=N-). ^13^C-NMR (δ, ppm): 21.4 (CH_3_); 69.10 (OCH_2_); 107.65 (C-8); 111.0 (C-6); 112.8 (C-3); 113.5 (C-9); 127.3 (C-6, Ar-); 127.8 (C-5); 129.4 (C-2, Ar-); 130.6 (C-5, Ar-); 131.4 (C-4, Ar-); 134.5 (C-3, Ar-); 135.2 (C-1, Ar-); 143.2 (C=N); 151.4 (C-9); 152.9 (C-4); 160.3 (C-7); 160.9 (C-2); 172.32 (CO-NH). Anal. Calcd. for C_19_H_15_ClN_2_O_4_ (370.79): C, 61.55; H, 4.08; N, 9.56. Found: C, 61.54; H, 4.07; N, 7.53.

*(E)-N^´^-(2,3-Dihydroxybenzylidene)-2-(4-methyl-2-oxo-2H-chromen-7-yloxy)acetohydrazide* (**2d**). M.p. 270–274 °C; yield (63%). FT-IR: ν_max_ 3,481; 3,436; 3,332; 3,280; 2,915; 1,719; 1,695; 1,620; 1,541; 1,473; 1,392; 1,265 and 1,153 cm^-1^. ^1^H-NMR (δ, ppm): 2.40 (s, 3H, CH_3_, C-4); 4.84 (s, 2H, CH_2_); 6.24 (s, 1H, H-3); 6.73 (s, 1H, H-8); 6.85 (d, 1H, H-6); 7.19 (d, 1H, H-5); 6.90-7.75 (m, 3H, arom.); 8.32 (s, 1H, NH); 8.52 (s, 1H, HC=N-); 9.27 (s, br., 1H, OH); 11.59 (s, br., 1H, OH). ^13^C-NMR (δ, ppm): 21.4 (CH_3_); 69.10 (OCH_2 _); 107.6 (C-8); 111.0 (C-6); 112.8 (C-3); 113.5 (C-9); 119.8 (C-4, Ar-); 120.1 (C-1, Ar-); 122.6 (C-5, Ar-); 123.5 (C-6, Ar-); 127.8 (C-5); 143.2 (C=N); 147.5 (C-3, Ar-); 151.4 (C-9); 151.8 (C-2, Ar-); 152.9 (C-4); 160.3 (C-7); 160.9 (C-2); 172.42 (CO-NH). Anal. Calcd. for C_19_H_16_N_2_O_6_ (368.34): C, 61.95; H, 4.38; N, 7.61. Found: C, 61.93; H, 4.36; N, 7.59. 

*(E)-N^´^-(2,4-Dihydroxybenzylidene)-2-(4-methyl-2-oxo-2H-chromen-7-yloxy)acetohydrazide* (**2e**). M.p. 261–262 °C; yield (48%). FT-IR: ν_max_ 3,357; 3,273; 3,169; 3,086; 2,924; 1,716; 1,671; 1,613; 1,512; 1,425; 1,391; 1,264 and 1,152 cm^-1^. ^1^H-NMR (δ, ppm): 2.40 (s, 3H, CH_3_, C-4); 4.84 (s, 2H, CH_2_); 6.24 (s, 1H, H-3); 6.73 (s, 1H, H-8); 6.85 (d, 1H, H-6); 7.20 (d, 1H, H-5); 6.90-7.75 (m, 3H, arom.); 8.32 (s, 1H, NH); 8.52 (s, 1H, HC=N-); 10.12 (s, br., 1H, OH); 11.40 (s, br., 1H, OH). ^13^C- NMR (δ, ppm): 21.4 (CH_3_); 69.10 (OCH_2 _); 103.5 (C-3, from Ph); 107.6 (C-8); 108.6 (C-5, Ar-); 111.0 (C-6); 111.3 (C-1, Ar-); 112.8 (C-3); 113.5 (C-9); 127.8 (C-5); 132.5 (C-6, Ar-); 143.2 (C=N); 151.4 (C-9); 152.9 (C-4); 160.3 (C-7); 160.9 (C-2); 162.4 (C-4, Ar-); 162.8 (C-2, Ar-); 172.40 (CO-NH). Anal. Calcd. for C_19_H_16_N_2_O_6_ (368.34): C, 61.95; H, 4.38; N, 7.61. Found: C, 61.93; H, 4.39; N, 7.59. 

*(E)-N^´^-(3,4-Dihydroxybenzylidene)-2-(4-methyl-2-oxo-2H-chromen-7-yloxy)acetohydrazide* (**2f**). M.p. 243–244 °C; yield (38%). FT-IR: ν_max_ 3,393; 3,239; 3,075; 3,071; 2,980; 1,716; 1,694; 1,612; 1,541; 1,509; 1,494; 1,393; 1,262 and 1,159 cm^-1^. ^1^H-NMR(δ, ppm): 2.40 (s, 3H, CH_3_, C-4); 4.84 (s, 2H, CH_2_); 6.24 (s, 1H, H-3); 6.73 (s, 1H, H-8); 6.85 (d, 1H, H-6); 7.20 (d, 1H, H-5); 6.90-7.75 (m, 3H, arom.); 8.32 (s, 1H, NH); 8.52 (s, 1H, HC=N-); 9.35 (s, br., 1H, OH); 10.14 (s, br., 1H, OH).^ 13^C-NMR (δ, ppm): 21.4 (CH_3_); 69.10 (OCH_2 _); 107.6 (C-8); 111.0 (C-6); 112.8 (C-3); 113.5 (C-9); 116.8 (C-2, Ar‑); 117.6 (C-5, Ar); 123.5 (C-6, Ar-); 127.3 (C-1, Ar-); 127.8 (C-5); 143.2 (C=N); 143.5 (C-3, Ar-); 149.8 (C-4, Ar-); 151.4 (C-9); 152.9 (C-4); 160.3 (C-7); 160.9 (C-2); 172.42 (CO-NH). Anal. Calcd. for C_19_H_16_N_2_O_6_ (368.34): C, 61.95; H, 4.38; N, 7.61. Found: C, 61.90; H, 4.35; N, 7.62.

*(E)-N^´^-(2,5-Dihydroxybenzylidene)-2-(4-methyl-2-oxo-2H-chromen-7-yloxy)acetohydrazide* (**2g**). M.p. 273–274 °C; yield (41%). IR: ν_max_ 3,418; 3,276; 3,075; 2,915; 1,705; 1,686; 1,658; 1,622; 1,611; 1,584; 1,550; 1,512; 1,428; 1,392; 1,288 and 1,149 cm^-1^. ^1^H-NMR (δ, ppm): 2.40 (s, 3H, CH_3_, C-4); 4.84 (s, 2H, CH_2_); 6.24 (s, 1H, H-3); 6.73 (s, 1H, H-8); 6.85 (d, 1H, H-6); 7.20 (d, 1H, H-5); 6.90-7.75 (m, 3H, arom.,); 8.32 (s, 1H, NH); 8.52 (s, 1H, HC=N-); 9.26 (s, br., 1H, OH); 11.93 (s, br., 1H, OH).^ 13^C-NMR (δ, ppm): 21.4 (CH_3_); 69.10 (OCH_2_); 107.6 (C-8); 111.0 (C-6); 112.8 (C-3); 113.5 (C-9); 116.5 (C-6, Ar-); 117.5 (C-3, Ar-); 119.4 (C-4, Ar-); 120.1 (C-1, Ar-); 127.8 (C-5); 143.2 (C=N); 151.4 (C-9); 151.3 (C-5, Ar-); 152.9 (C-4); 153.8 (C-2, Ar-); 160.3 (C-7); 160.8 (C-2); 172.44 (CO-NH). Anal. Calcd. for C_19_H_16_N_2_O_6_ (368.34): C, 61.95; H, 4.38; N, 7.61. Found: C, 61.92; H, 4.37; N, 7.62.

*(E)-N^´^-(4-Hydroxy-3-methoxybenzylidene)-2-(4-methyl-2-oxo-2H-chromen-7-yloxy) acetohydrazide* (**2h**) [5]. M.p. 253–254 °C; yield (76%). FT-IR: ν_max_ 3,530; 3,481; 3,319; 3,268; 2,914; 1,720; 1,695; 1,618; 1,540; 1,472; 1,390; 1,265 and 1,153 cm^-1^. ^1^H-NMR (δ, ppm): 2.40 (s, 3H, CH_3_, C-4); 3.82 (3H, s, OCH_3_); 4.84 (s, 2H, OCH_2_); 6.24 (s, 1H, H-3); 6.73 (s, 1H, H-8); 6.85 (d, 1H, H-6); 7.20 (d, 1H, H-5); 6.90-7.75 (m, 3H, arom.); 8.32 (s, 1H, NH); 8.52 (s, 1H, HC=N-); 11.50 (s, br., 1H, OH). ^13^C-NMR (δ, ppm): 21.4 (CH_3_); 56.2 (OCH_3_); 69.10 (OCH_2 _); 107.6 (C-8);); 111.0 (C-6); 112.8 (C-3); 113.5 (C‑9); 114.8 (C-2, Ar-); 117.3 (C-5, Ar-); 123.0 (C-6, Ar-); 127.2 (C-1, Ar-); 127.8 (C-5); 143.2 (C=N); 148.1 (C-4, Ar-); 151.4 (C-9); 151.5 (C-3, Ar-); 152.9 (C-4); 160.3 (C-7); 160.7 (C-2); 172.42 (CO-NH). Anal. Calcd. for C_20_H_18_N_2_O_6_ (382.37): C, 62.82; H, 4.74; N, 7.33. Found: C, 62.79; H, 4.73; N, 7.31.

*(E)-N´-(3-Phenoxybenzylidene)-2-(4-methyl-2-oxo-2H-chromen-7-yloxy)-acetohydrazide* (**2i**). M.p. 178–180 °C; yield (72%). FT-IR: ν_max_ 3,526; 3,476; 3,329; 3,288; 2,916; 1,723; 1,690; 1,623; 1,544; 1,470; 1,392; 1,265 and 1,153 cm^-1^. ^1^H-NMR (δ, ppm): 2.40 (s, 3H, CH_3_, C-4); 4.84 (s, 2H, CH_2_); 6.24 (s, 1H, H-3); 6.73 (s, 1H, H-8); 6.85 (d, 1H, H-6); 7.20 (d, 1H, H-5); 6.90-7.75 (m, 9H, arom.,); 8.32 (s, 1H, NH); 8.52 (s, 1H, HC=N-). ^13^C-NMR (δ, ppm): 21.4 (CH_3_); 56.2 (OCH_3_); 69.10 (OCH_2_); 107.6 (C-8); 111.0 (C-6); 112.8 (C-3); 113.5 (C-9); 116.8 (C-2, Ar-Ph); 117.4 (C-2,6, Ar- PhO); 119.7 (C-4, Ar- Ph); 121.8 (C-4, Ar-PhO); 122.3 (C-6, Ar- Ph); 127.8 (C-5); 128.5 (C-3,5, Ar-PhO); 128.8 (C-5, Ar- Ph); 133.6 (C-1, Ar- Ph); 143.2 (C=N); 151.4 (C-9); 152.9 (C-4); 157.1 (C-1, Ar- PhO); 157.3 (C-3, Ar- Ph); 160.3 (C-7); 160.8 (C-2); 172.40 (CO-NH). Anal. Calcd. for C_25_H_20_N_2_O_5_ (428.44): C, 70.08; H, 4.71; N, 6.54. Found: C, 70.10; H, 4.72; N, 6.55.

*(E)-N´-(4-(Dimethylamino)benzylidene)-2-(4-methyl-2-oxo-2H-chromen-7-yloxy) acetohydrazide* (**2j**) [6]. M.p. 260–262 °C; yield (86%). FT-IR: ν_max_ 3,528; 3,471; 3,329; 3,285; 2,912; 1,721; 1,695; 1,621; 1,541; 1,473; 1,392; 1,265 and 1,153 cm^-1^. ^1^H-NMR (δ, ppm): 2.40 (s, 3H, CH_3_, C-4); 2.86 (s, 6H, N(CH_3_)_2_); 4.84 (s, 2H, CH_2_); 6.24 (s, 1H, H-3); 6.73 (s, 1H, H-8); 6.85 (d, 1H, H-6); 7.20 (d, 1H, H-5); 6.90-7.75 (m, 4H, arom.); 8.32 (s, 1H, NH); 8.52 (s, 1H, HC=N-) ^13^C-NMR, (δ, ppm): 21.4 (CH_3_); 41.4 (N(CH _3 _)_2_); 69.10 (OCH_2 _); 107.6 (C-8); 111.0 (C-6); 112.8 (C-3); 113.5 (C-9); 114.5 (C-3,5, Ar‑); 123.04 (C-1, Ar-); 127.8 (C-5); 130.2 (C-2,6, Ar-); 143.2 (C=N); 151.4 (C-10); 151.8 (C-4, Ar-); 152.8 (C-4,coum.); 160.3 (C-7); 160.9 (C-2); 172.42 (CO-NH). Anal. Calcd. For C_21_H_21_N_3_O_4_ (379.41): C, 66.48; H, 5.58; N, 11.08. Found: C, 66.50; H, 5.57; N, 11.10. 

*(E)-N´-(2-Hydroxy-5-nitrobenzylidene)-2-(4-methyl-2-oxo-2H-chromen-7-yloxy)acetohydrazide* (**2k**). M.p. 288–290 °C; yield (90%). FT-IR: ν_max_ 3,536; 3,481; 3,329; 3,268; 2,910; 1,724; 1,690; 1,622; 1,539; 1,474; 1,390; 1,265 and 1,153 cm^-1^. ^1^H NMR (δ, ppm): 2.40 (s, 3H, CH_3_, C-4); 4.84 (s, 2H, CH_2_); 6.24 (s, 1H, H-3); 6.73 (s, 1H, H-8); 6.85 (d, 1H, H-6); 7.20 (d, 1H, H-5); 6.90-7.75 (m, 3H, arom.); 8.32 (s, 1H, NH); 8.52 (s, 1H, HC=N-); 11.59 (s, br., 1H, OH).^ 13^C-NMR (δ, ppm): 21.4 (CH_3_); 69.10 (OCH_2_); 107.6 (C-8); 111.0 (C-6); 112.8 (C-3); 113.5 (C-9); 117.1 (C-3, Ar-); 119.6 (C-1, Ar-); 120.1 (C-1, Ar-); 124.5 (C-4, Ar-); 125.7 (C-6, Ar-); 127.8 (C-5); 141.3 (C-5, Ar-); 143.2 (C=N); 151.4 (C-9); 152.9 (C-4); 160.3 (C-7); 160.8 (C-2); 167.8 (C-2, Ar-); 172.41 (CO-NH). Anal. Calcd. for C_19 _H_15_N_3_O_7_ (397.34): C, 57.43 ; H, 3.81; N,10.58. Found: C, 57.40; H, 3.82; N, 10.54. 

*(E)-2-(4-Methyl-2-oxo-2H-chromen-7-yloxy)-N´-[(E)-3-phenylallylidene]acetohydrazide* (**2l**) [6]. M.p. 249–250 °C; yield (46%). FT-IR: ν_max_ 3,530; 3,481; 3,339; 3,288; 2,916; 1,720; 1,695; 1,621; 1,541; 1,473; 1,392; 1,265 and 1,153 cm^-1^. ^1^H-NMR (δ, ppm): 2.40 (s, 3H, CH_3_, C-4); 4.84 (s, 2H, CH_2_); 5.72 (d, 1H, styryl); 6.24 (s, 1H, H-3); 6.63 (d, 1H, styryl); 6.73 (s, 1H, H-8); 6.85 (d, 1H, H-6); 7.20 (d, 1H, H-5); 6.90-7.75 (m, 3H, arom.); 8.32 (s, 1H, NH); 8.52 (s, 1H, HC=N-).^ 13^C-NMR (δ, ppm): 21.4 (CH_3_); 69.10 (OCH_2_); 107.6 (C-8); 111.0 (C-6); 112.8 (C-3); 113.5 (C-9); 126.1 (C-1, Ar-ethenyl); 126.6 (C-2,6, Ar-); 127.8 (C-5); 128.5 (C-4, Ar-); 128.9 (C-3,5, Ar-); 137.2 (C=N); 139.2 (C-2, Ar-ethenyl); 151.4 (C-10); 152.9 (C-4); 160.3 (C-7); 161.0 (C-2); 172.42 (CO-NH). Anal. Calcd. For C_21_H_18_N_2_O_4_ (362.38): C, 69.60; H, 5.01; N, 7.73. Found. C, 69.59; H, 4.99; N, 7.70. 

### 3.3. General procedure for preparation of N-(2-(substituted)-4-oxothiazolidin-3-yl)-2-(4-methyl-2-oxo-2H-chromen-7-yloxy) acetamides *3a-l* [33]

To a solution of (*E*)-2-(4-methyl-2-oxo-2*H*-chromen-7-yloxy)*-N*´-[(*E*)-3-arylidene]acetohydrazide **2a-l** (0.01 mol) in dry 1,4-dioxane (15 mL), freshly distilled mercaptoacetic acid (0.01 mol) and anhydrous ZnCl_2_ (0.1 g) were added and the mixture was heated under reflux 10 to 12 hours. The solvent was removed (reduced pressure) and residue washed with 5% sodium bicarbonate solution (3 × 20 mL) and water (2 × 20 mL), dried, and recrystallized from an appropriate solvent.

*N-(2-(2-Hydroxyphenyl)-4-oxothiazolidin-3-yl)-2-(4-methyl-2-oxo-2H-chromen-7-yloxy) acetamide* (**3a**). M.p. 284–286 °C; yield (72%). FT-IR: ν_max_ 3,418; 3,182; 3,100; 2,906; 2,853; 1,725; 1,681; 1,623; 1,527; 1,489; 1,393; 1,300 and 1,144 cm^-1^. ^1^H-NMR (δ, ppm): 2.40 (s, 3H, CH_3, _C-4); 3.34 (s, 2H, COCH_2_S); 4.84 (s, 2H, OCH_2_); 5.28 (s, 1H, SCHN); 6.24 (s, 1H, C-3); 6.87 (s, 1H, H-8), 6.98 (d, 1H, H-6); 7.25–7.75 (m, 4H, arom.); 8.06 (d, 1H, H-5); 8.33(s, 1H, NH); 10.99 (s, br.,.1H, OH). ^13^C- NMR (δ, ppm): 21.4 (CH_3_); 32.92 (COCH_2_S); 69.10 (OCH_2_); 107.65 (C-8); 111.0 (C-6); 112.8 (C-3); 113.6 (C-10); 116.1 (C-3, Ar-); 118.4 (C-3, Ar-); 121.7 (C-5, Ar-); 127.8 (C-5); 130.5 (C-6, Ar-); 132.4 (C-1, Ar-); 161.2 (C-2, Ar-); 143.2 (CO-N); 151.4 (C-9); 152.9 (C-4); 160.3 (C-7); 160.9 (C-4); 172.45 (CO-NH). Anal. Calcd. for C_21_H_18_N_2_O_6_S (426.44), C, 59.15; H, 4.25; N, 6.57; S, 7.52. Found: C, 59.10; H, 4.27; N, 6.54; S, 7.49. 

*N-(2-(2-Chlorophenyl)-4-oxothiazolidin-3-yl)-2-(4-methyl-2-oxo-2H-chromen-7-yloxy) acetamide* (**3b**) [26]. M.p. 248–250 °C; yield (82%). FT-IR: ν_max_ 3,280; 2,940; 2,854; 1,722; 1,710; 1,621; 1,580; 1,515; 1,394; 1,295 and 1,146 cm^-1^. ^1^H-NMR (δ, ppm): 2.40 (s, 3H, CH_3_, C-4); 3.35 (s, 2H, COCH_2_S); 4.84 (s, 2H, OCH_2_); 5.29 (s, 1H, SCHN); 6.22 (s, 1H, H-3); 6.99 (s, 1H, H-8); 7.05 (d, 1H, H-6); 7.39–7.75 (m, 4H, arom.); 8.06 (d, 1H, H-5); 8.41 (s, 1H, NH). ^13^C-NMR (δ, ppm): 21.4 (CH_3_); 32.92 (COCH_2_S); 47.81 (SCHN); 69.10 (OCH_2_); 107.65 (C-8); 111.0 (C-6); 112.8 (C-3); 113.4 (C-10); 127.1 (C-5, Ar-); 127.8 (C-5); 129.1 (C-3, Ar-); 130.4 (C-6, Ar-); 132.5 (C-4, Ar-); 133.2 (C-1, Ar-); 134.2 (C-2, Ar-); 143.2 (CO-N); 151.2 (C-19); 152.9 (C-4); 160.3 (C-7); 160.9 (C-2); 172.42 (CO-NH). Anal. Calcd. for C_21_H_17_Cl N_2_O_5_S (444.89), C, 56.69; H, 3.85; N, 6.30; S, 7.21. Found: C, 56.70; H, 3.83; N, 6.31; S, 7.20.

*N-(2-(3-Chlorophenyl)-4-oxothiazolidin-3-yl)-2-(4-methyl-2-oxo-2H-chromen-7-yloxy) acetamide *(**3c**). M.p. 242–244 °C; yield (86%). FT-IR: ν_max_ 3,438; 3,200; 3,112; 2,987; 1,723; 1,688; 1,627; 1,516; 1,430; 1,390; 1,264 and 1,136 cm^-1^. ^1^H-NMR (δ, ppm): 2.40 (s, 3H, CH_3, _C-4); 3.38 (s, 2H, COCH_2_S); 4.82 (s, 2H, CH_2_); 5.28 (s, 1H, SCHN); 6.23 (s, 1H, H-3); 6.98 (s, 1H, H-8); 7.02 (d, 1H, H-6); 7.41–7.74 (m, 4H, arom.); 8.09 (d, 1H, H-5); 8.40 (s, 1H, NH). ^13^C-NMR (δ, ppm): 21.4 (CH_3_); 32.92 (COCH_2_S); 47.81 (SCHN); 69.10 (OCH_2_); 107.65 (C-8); 111.0 (C-6); 112.8 (C-3); 113.5 (C-10); 127.3 (C-6, Ar-); 127.8 (C-5); 129.4 (C-2, Ar-); 130.6 (C-5, Ar-); 131.4 (C-4, Ar-); 134.5 (C-3, Ar-); 135.2 (C-1, Ar-); 143.3 (CO-N); 151.4 (C-9); 152.9 (C-4); 160.3 (C-7); 160.9 (C-2); 172.42 (CO-NH). Anal. Calcd. for C_21_H_17_Cl N_2_O_5_S (444.89), C, 56.69; H, 3.85; N, 6.30; S, 7.21.Found: C, 56.62; H, 3.82; N, 6.31; S, 7.24.

*N-(2-(2,3-Dihydroxyphenyl)-4-oxothiazolidin-3-yl)-2-(4-methyl-2-oxo-2H-chromen-7-yloxy)* *acetamide* (**3d**). M.p. 270–271 °C; yield (63%). FT-IR: ν_max_ 3,531; 3,471; 3,309; 3,268; 2,926; 1,725; 1,698; 1,626; 1,551; 1,483; 1,388; 1,260 and 1,143 cm^-1^. ^1^H-NMR (δ, ppm): 2.40 (s, 3H, CH_3_, C-4); 3.34 (s, 2H, COCH_2_S); 4.84 (s, 2H, CH_2_); 5.28 (s, 1H, SCHN); 6.24 (s, 1H, H-3); 6.73 (s, 1H, H-8); 6.85 (d, 1H, H-6); 7.20 (d, 1H, H-5); 6.90–7.75 (m, 3H, arom.); 8.32 (s, 1H, NH); 9.27 (s, br., 1H, OH); 11.59 (s, br., 1H, OH).^ 13^C-NMR (δ, ppm): 21.4 (CH_3_); 32.92 (COCH_2_S); 47.81 (SCHN); 69.10 (OCH_2_); 107.6 (C-8); 111.0 (C-6); 112.8 (C-3); 113.5 (C-10); 119.8 (C-4, Ar-); 120.1 (C-1, Ar-); 122.6 (C-5, Ar-); 123.5 (C-6, Ar-); 127.8 (C-5); 143.2 (CO-N); 147.5 (C-3, Ar-); 151.4 (C-9); 151.8 (C-2, Ar-); 152.9 (C-4); 160.3 (C-7); 160.9 (C-2); 172.43 (CO-NH). Anal. Calcd. for C_21_H_18_N_2_O_7_S (442.44), C, 57.01; H, 4.10; N, 6.33; S, 7.25. Found: C, 57.00; H, 4.08; N, 6.30; S, 7.23. 

*N-(2-(2,4-Dihydroxyphenyl)-4-oxothiazolidin-3-yl)-2-(4-methyl-2-oxo-2H-chromen-7-yloxy) acetamide* (**3e**). M.p. 261–262 °C; yield (48%). FT-IR: ν_max_ 3,345; 3,282; 3,172; 3,106; 2,912; 1,726; 1,691; 1,622; 1,510; 1,430; 1,382; 1,274 and 1,141 cm^-1^. ^1^H NMR (δ, ppm): 2.40 (s, 3H, CH_3_, C-4); 3.38 (s, 2H, COCH_2_S); 4.84 (s, 2H, CH_2_); 5.29 (s, 1H, SCHN); 6.24 (s, 1H, H-3); 6.73 (s, 1H, H-8); 6.85 (d, 1H, H-6); 7.20 (d, 1H, H-5); 6.90–7.70 (m, 3H, arom.); 8.32 (s, 1H, NH); 10.12 (s, br., 1H, OH); 11.40 (s, br., 1H, OH).^ 13^C-NMR (δ, ppm): 21.4 (CH_3_); 32.92 (COCH_2_S); 47.81 (SCHN); 69.30 (OCH_2_); 102.7 (C-3, Ar-); 107.9 (C-8); 108.6 (C-5, Ar-); 111.5 (C-6); 111.7 (C-1, Ar-); 112.8 (C-3); 113.6 (C-10); 127.0 (C-5); 132.5 (C-6, Ar-); 143.2 (CO-N); 151.4 (C-9); 152.9 (C-4); 160.4 (C-7); 160.9 (C-2); 162.4 (C-4, Ar-); 162.8 (C-2, Ar-); 172.43 (CO-NH). Anal. Calcd. for C_21_H_18_N_2_O_7_S (442.44), C, 57.01; H, 4.10; N, 6.33; S, 7.25. Found: C, 57.04; H, 4.97; N, 6.32; S, 7.29. 

*N-(2-(3,4-Dihydroxyphenyl)-4-oxothiazolidin-3-yl)-2-(4-methyl-2-oxo-2H-chromen-7-yloxy) acetamide* (**3f**). M.p. 243–244 °C; yield (38%). FT-IR: ν_max_ 3,389; 3,209; 3,045; 3,037; 2,974; 1,726; 1,698; 1,612; 1,548; 1,521; 1,489; 1,396; 1,262 and 1,150 cm^-1^. ^1^H-NMR (δ, ppm): 2.40 (s, 3H, CH_3_, C-4); 3.35 (s, 2H, COCH_2_S); 4.84 (s, 2H, CH_2_); 5.28 (s, 1H, SCHN); 6.24 (s, 1H, H-3); 6.73 (s, 1H, H-8); 6.85 (d, 1H, H-6); 7.20 (d, 1H, H-5); 6.90-7.65 (m, 3H, arom.); 8.32 (s, 1H, NH); 9.35 (s, br., 1H, OH); 10.14 (s, br., 1H, OH).^ 13^C-NMR (δ, ppm): 21.4 (CH_3_); 32.92 (COCH_2_S); 47.81 (SCHN); 69.10 (OCH_2_); 107.6 (C-8); 111.0 (C-6); 112.8 (C-3); 113.5 (C-10); 116.8 (C-2, Ar-); 117.6 (C-5, Ar-); 123.5 (C-6, Ar-); 127.3 (C-1, Ar-); 127.8 (C-5); 143.2 (C-N); 143.5 (C-3, Ar-); 149.8 (C-4, Ar-); 151.4 (C‑9); 152.9 (C-4); 160.3 (C-7); 160.9 (C-2); 172.42 (CO-NH). Anal. Calcd. for C_21_H_18_N_2_O_7_S (442.44), C, 57.01; H, 4.10; N, 6.33; S, 7.25. Found: C, 56.99; H, 3.89; N, 6.32; S, 7.24.

*N-(2-(2,5-Dihydroxyphenyl)-4-oxothiazolidin-3-yl)-2-(4-methyl-2-oxo-2H-chromen-7-yloxy) acetamide* (**3g**). M.p. 278–279 °C; yield (41%). FT-IR: ν_max_ 3,420; 3,281; 3,065; 2,915; 1,708; 1,716; 1,688; 1,648; 1,627; 1,581; 1,554; 1,521; 1,418; 1,390; 1,278 and 1,149 cm^-1^. ^1^H-NMR (δ, ppm): 2.40 (s, 3H, CH_3_, C-4); 3.38 (s,2H,COCH_2_S); 4.84 (s, 2H, CH_2_); 5.28 (s, 1H, SCHN); 6.24 (s, 1H, H-3); 6.73 (s, 1H, H-8); 6.85 (d, 1H, H-6); 7.20 (d, 1H, H-5); 6.90-7.72 (m, 3H, arom.); 8.32 (s, 1H, NH); 9.26 (s, br., 1H, OH); 11.93 (s, br., 1H, OH).^ 13^C-NMR (δ, ppm): 21.4 (CH_3_); 32.92 (COCH_2_S); 47.81 (SCHN); 69.10 (OCH_2_); 107.6 (C-8); 111.0 (C-6); 112.8 (C-3); 113.5 (C-10); 116.5 (C-6, Ar-); 117.5 (C-3, Ar-); 119.4 (C-4, Ar-); 120.1 (C-1, Ar-); 127.8 (C-5); 143.2 (C-N); 151.4 (C-9); 151.3 (C-5, Ar‑); 152.9 (C-4); 153.8 (C-2, Ar-); 160.3 (C-7); 160.9 (C-2); 172.43 (CO-NH). Anal. Calcd. for C_21_H_18_N_2_O_7_S (442.44), C, 57.01; H, 4.10; N, 6.33; S, 7.25. Found: C, 57.00; H, 4.08; N, 6.31; S, 7.20.

*N-(2-(4-hydroxy-3-methoxyphenyl)-4-oxothiazolidin-3-yl)-2-(4-methyl-2-oxo-2H-chromen-7-yloxy) acetamide* (**3h**) [23]. M.p. 233–234 °C; yield (76%). FT-IR: ν_max_ 3,517; 3,478; 3,329; 3,274; 2,915; 1,723; 1,698; 1,629; 1,536; 1,469; 1,393; 1,255 and 1,145 cm^-1^. ^1^H-NMR (δ, ppm): 2.40 (s, 3H, CH_3_, C-4); 3.37 (s, 2H, COCH_2_S); 3.76 (s, 3H, OCH_3_); 4.84 (s, 2H, CH_2_); 5.27 (s, 1H, SCHN); 6.24 (s, 1H, H-3); 6.73 (s, 1H, H-8); 6.85 (d, 1H, H-6); 7.20 (d, 1H, H-5); 6.90-7.71 (m, 3H, arom.); 8.32 (s, 1H, NH); 11.59 (s, br., 1H, OH). ^13^C-NMR (δ, ppm): 21.4 (CH_3_); 32.92 (COCH_2_S); 47.81 (SCHN); 56.2 (OCH_3_); 69.10 (OCH_2_); 107.6 (C-8); 111.0 (C-6); 112.8 (C-3); 113.5 (C-10); 114.8 (C-2, Ar-); 117.3 (C-5, Ar-); 123.0 (C-6, Ar-); 127.2 (C-1, Ar-); 127.8 (C-5); 143.2 (C-N); 148.1 (C-4, Ar-); 151.4 (C‑9); 151.5 (C-3, Ar-); 152.9 (C-4); 160.3 (C-7); 160.9 (C-2); 172.40 (CO-NH). Anal. Calcd. for C_22_H_20_N_2_O_7_S (456.47), C, 57.89; H, 4.42; N, 6.14; S, 7.02. Found: C, 57.88; H, 4.39; N, 6.13; S, 7.01.

*2-(4-Methyl-2-oxo-2H-chromen-7-yloxy)-N-(4-oxo-2-(3-phenoxyphenyl)thiazolidin-3-yl) acetamide* (**3i**). M.p. 217–218 °C; yield (72%). FT-IR: ν_max_ 3,500; 3,471; 3,329; 3,279; 2,910; 1,720; 1,697; 1,619; 1,531; 1,467; 1,392; 1,264 and 1,151 cm^-1^. ^1^H-NMR (δ, ppm): 2.40 (s, 3H, CH_3_, C-4); 3.38 (s, 2H, COCH_2_S); 4.84 (s, 2H, CH_2_); 5.29 (s, 1H, SCHN); 6.24 (s, 1H, H-3); 6.73 (s, 1H, H-8); 6.85 (d, 1H, H-6); 7.20 (d, 1H, H-5); 6.90-7.76 (m, 9H, arom.); 8.32 (s, 1H, NH). ^13^C-NMR (δ, ppm), 21.4 (CH_3_); 32.92 (COCH_2_S); 47.81 (SCHN); 69.10 (OCH_2_); 107.6 (C-8); 111.0 (C-6); 112.8 (C-3); 113.5 (C-10); 115.7 (C-4, Ar-Ph); 116.8 (C-2, Ar- Ph); 117.4 (C-2,6, Ar- PhO); 121.8 (C‑4, Ar- PhO); 122.3 (C-6, Ar- Ph); 127.8 (C-5); 128.5 (C-3,5, Ar- PhO); 128.8 (C-5, Ar- Ph); 133.6 (C-1, Ar- Ph); 151.4 (C-9); 152.9 (C-4); 157.1 (C-1, Ar- PhO); 157.3 (C-3, Ar- Ph); 160.3 (C-7); 160.9 (C-2); 166.1 (CO-NH); 168.2 (CO-N). Anal. Calcd. for C_27_H_22_N_2_O_6_S (502.54), C, 64.53; H, 4.41; N, 5.57; S, 6.38. Found: C, 64.50; H, 4.39; N, 5.53; S, 6.39.

*N-(2-(4-(Dimethylamino)phenyl)-4-oxothiazolidin-3-yl)-2-(4-methyl-2-oxo-2H-chromen-7-yloxy)-acetamide* (**3j**). M.p. 227–228 °C; yield (76%). FT-IR: ν_max_ 3,526; 3,473; 3,329; 3,269; 2,926; 1,729; 1,690; 1,624; 1,539; 1,473; 1,378; 1,260 and 1,150 cm^-1^. ^1^H-NMR (δ, ppm): 2.40 (s, 3H, CH_3_, C-4); 2.86 (s, 6H, N(CH_3_)_2_); 3.36 (s, 2H, COCH_2_S); 4.84 (s, 2H, CH_2_); 5.29 (s, 1H, SCHN); 6.24 (s, 1H, H-3); 6.73 (s, 1H, H-8); 6.85 (d, 1H, H-6); 7.20 (d, 1H, H-5); 6.90-7.7 (m, 4H, arom.); 8.32 (s, 1H, NH). ^13^C-NMR (δ, ppm): 21.4 (CH_3_); 32.92 (COCH_2_S); 41.4(N(CH_3_)_2_); 47.81 (SCHN); 69.10 (OCH_2_); 107.6 (C-8); 111.0 (C-6); 112.8 (C-3); 113.5 (C-10); 114.5 (C-3,5, Ar-); 123.04 (C-1, Ar); 130.2 (C‑2,6, Ar); 143.2 (C-N); 151.4 (C-9); 151.8 (C-4, Ar-); 160.2 (C-7); 160.9 (C-2); 172.43 (CO-NH). Anal. Calcd. for C_23_H_23_N_3_O_5_S (453.51), C, 60.91; H, 5.11; N, 9.27; S, 7.07. Found: C, 60.92; H, 5.09; N, 9.25; S, 7.01. 

*N-(2-(2-Hydroxy-5-nitrophenyl)-4-oxothiazolidin-3-yl)-2-(4-methyl-2-oxo-2H-chromen-7-yloxy) acetamide* (**3k**). M.p. 288–290 °C; yield (90%). FT-IR: ν_max_ 3,468; 3,459; 3,329; 3,218; 2,910; 1,720; 1,692; 1,625; 1,536; 1,469; 1,386; 1,260 and 1,147 cm^-1^. ^1^H-NMR (δ, ppm): 2.40 (s, 3H, CH_3_, C-4); 3.37 (s, 2H, COCH_2_S); 4.84 (s, 2H, CH_2_); 5.28 (s, 1H, SCHN); 6.24 (s, 1H, H-3); 6.73 (s, 1H, H-8); 6.85 (d, 1H, H-6); 7.20 (d, 1H, H-5); 6.90–7.82 (m, 3H, arom.); 8.32 (s, 1H, NH); 11.54 (s, br., 1H, OH).^ 13^C-NMR (δ, ppm): 21.4 (CH_3_); 32.92 (COCH_2_S); 47.81 (SCHN); 69.10 (OCH_2_); 107.6 (C-8); 111.0 (C-6); 112.8 (C-3); 113.5 (C-10); 117.1 (C-3, from Ph); 120.1 (C-1, from Ph); 124.5 (C-4, from Ph); 125.7 (C-6, from Ph); 127.8 (C-5); 141.3 (C-5, from Ph); 143.3 (C-N); 151.4 (C-9); 152.9 (C-4); 160.3 (C-7); 160.9 (C-2); 167.8 (C-2, from Ph); 174.0 (CO-NH). Anal. Calcd. for C_21_H_17_N_3_O_8_S (471.44), C, 53.50; H, 3.63; N, 8.91; S, 6.80. Found: C, 53.47; H, 3.64; N, 8.90; S, 6.79.

*(E)-2-(4-Methyl-2-oxo-2H-chromen-7-yloxy)-N-(4-oxo-2-styrylhiazolidin-3-yl)acetamide *(**3l**). M.p. 249–250 °C; yield (46%). FT-IR: ν_max_ 3,489; 3,416; 3,309; 3,292; 2,906; 1,728; 1,695; 1,621; 1,543; 1,468; 1,389; 1,254 and 1,131 cm^-1^. ^1^H-NMR (δ, ppm): 2.40 (s, 3H, CH_3_, C-4); 3.36 (s, 2H, COCH_2_S); 4.84 (s, 2H, CH_2_); 5.26 (s, 1H, SCHN); 6.13 (d, 1H, styryl); 6.24 (s, 1H, H-3); 6.41 (d, 1H, styryl); 6.73 (s, 1H, H-8); 6.85 (d, 1H, H-6); 7.20 (d, 1H, H-5); 6.94–7.70 (m, 5H, arom.); 8.32 (s, 1H, NH).^ 13^C-NMR (δ, ppm) 21.4 (CH_3_); 32.92 (COCH_2_S); 47.81 (SCHN); 69.10 (OCH_2_); 107.6 (C-8); 111.0 (C-6); 112.8 (C-3); 113.5 (C-9); 126.1 (C-1, styryl); 126.6 (C-2,6, Ar-); 127.8 (C-5); 128.5 (C-4, Ar-); 128.9 (C-3,5, Ar-); 137.2 (C-N); 139.2 (C-2, styryl); 151.4 (C-9); 152.9 (C-4); 160.3 (C-7); 160.9 (C‑2); 174.4 (CO-NH). Anal. Calcd. for C_23_H_20_N_2_O_5_S (436.48), C, 63.29; H, 4.62; N, 6.42; S, 7.35. Found: C, 63.27; H, 4.61; N, 6.39; S, 7.31.

### 3.4. Evaluation of antioxidant activity

The antioxidant activity of tested coumarin derivatives was evaluated by the phosphomolybdenum method according to the procedure in [34]. This method is based on the reduction of Mo(VI) to Mo(V) by the tested compounds followed by formation of a green phosphate/Mo(V) complex at acid pH. An aliquot of sample solution (100 μL, 2 mM in DMSO) is mixed with the reagent solution (1 mL, 0.6 M sulphuric acid, 28 mM sodium phosphate and 4 mM ammonium molybdate). The samples are incubated in a water bath at 95 °C for 90 minutes. Samples are cooled to room temperature and the absorbance was measured at 695 nm. The antioxidant activity was expressed relative to the antioxidant activity of same concentration of ascorbic acid.

## 4. Conclusions

In this study a series of Schiff’s bases (*E*)-*N*-2-aryliden-2-(4-methyl-2-oxo-2*H*-chromen-7-yloxy)acetohydrazides **2a-l** and novel N-(2-(substituted phenyl)-4-oxo-thiazolidin-3-yl)-2-(4-methyl-2-oxo-2*H*-chromen-7-yloxy)acetamides **3a-l **were synthesized and evaluated for their antioxidant activity by phosphomolybdenum method. The 1,3-thiazolidine-4-one derivatives containing the coumarin moiety were synthesized by cyclocondensation of the Schiff's bases and mercaptoacetic acid. Compounds which are indicated as already known are resynthetized and the analytical data obtained for these compounds were comparable, but slightly different from those of other authors. For all the novel compounds structures were elucidated by the means of various spectral methods. Two of the Schiff’s bases (**2g, 2d**) and three of 1,3-thiazolidine-4-ones (**3g, 3d, 3e**) proved to have better antioxidant activity in comparison with ascorbic acid. In conclusion, it is evident that the substituents on the phenyl ring have a great influence on antioxidant activity.
